# American Pharmaceutical Association

**Published:** 1875-10

**Authors:** 

**Affiliations:** Boston


### AMERICAN PHARMACEUTICAL ASSOCIATION.

The twenty-third annual meeting of this organization, recently held at Boston, was, on the whole, the most successful in the history of the Society. The objects of the Association commend themselves to the favorable notice of physicians and the public, as well as those more directly interested in the drug business, aiming as it does to improve the drug market by detecting and exposing inferior or adulterated drugs, to advance the science and art of pharmacy, and encourage a higher state of professional feeling, discouraging empiricism and humbug, and promoting proper relations between the druggist or pharmacist, the physician, and the public. To promote these and similar ends, committees are appointed to report upon the progress of pharmacy, upon the drug market, upon adulterations of drugs, on pharmaceutical legislation, and on all such topics as may be of importance to the profession in general. Of course, the main interest centres in the annual meetings, when these reports are presented, and papers read in answer to queries previously proposed, together with volunteer communications on kindred subjects, the whole being afterwards published in a volume of "Proceedings." At the recent meeting in Boston, nearly 500 members and ladies were in attendance, and many of the papers presented were of unusual interest. Among them may be mentioned one on the relative merits of the various drug mills in the market, on the solubility of sugar-coated and other pills, on the so-called tasteless preparations of iron introduced by Mr. Creuse, on phosphuretted resin, on the antiseptic properties of chloral, on suppository moulds, on Mezquite gum, on the true botanical source of Matico, on the composition of the nostrum called Vinegar Bitters, and three especially interesting essays on the preparation of dilute phosphoric acid by the second process of the United States Pharmacopœia, in which the fact was developed that all the glacial phosphoric acid of the market is contaminated with phosphate of sodium, sometimes to the extent of 25 per cent., without which it is impossible to produce a handsome, glassy acid that will not deliquesce. These few are mentioned almost at random, out of about fifty papers presented, in order to show the range of subjects that are brought before the Association, and the value of the information imparted, often as the result of very considerable research on the part of the writers. The Association numbers over 1100 members, including most of the leading men in the business, as well as many less distinguished, who are willing to share in its advantages, and to work for its high ends. The membership is distributed over all the States of the Union and Canada, with some few even in the West Indies and South America, the largest numbers naturally being found in New York, Pennsylvania, and Massachusetts. Elmira is represented by your well-known druggist, M. S. Bidwell. As the standard of education and of professional spirit in the drug business is raised, this Association increasingly prospers, the additions at this last meeting being larger than ever before. It is certainly worthy of the patronage of every intelligent and fair-minded pharmacist in the land. The next meeting, like everything else that year, will of course be held in Philadelphia.

BOSTON, Sept. 15th, 1875. PHARMACIST.

